# Development of the Mental Health Peer Support Questionnaire in colleges and vocational schools in Singapore

**DOI:** 10.1186/s13033-022-00555-6

**Published:** 2022-09-02

**Authors:** QianHui Ma, Joseph J. Gallo, Jeanine M. Parisi, Jin Hui Joo

**Affiliations:** 1 Department of Mental Health, Johns Hopkins University Bloomberg School of Public Health, 624 North Broadway Street, 8Th Floor, Baltimore, MD 21205 USA; 2 Department of Mental Health, Johns Hopkins University Bloomberg School of Public Health, 624 North Broadway Street, Baltimore, MD 21205 USA; 3 Department of Mental Health, Johns Hopkins University Bloomberg School of Public Health, 624. North Broadway Street, Baltimore, MD 21205 USA; 4 Department of Psychiatry and Behavioral Sciences, Johns Hopkins University School of Medicine, Johns Hopkins Bayview Medical Center, 5300 Alpha Commons Dr, Baltimore, MD 21224 USA

**Keywords:** Peer support, Mental health, Scale development, Emerging adulthood, School program

## Abstract

**Background:**

A nation-wide mental health peer support initiative was established in college and vocational schools in Singapore. The purpose of this cross-sectional study was to develop and validate a 20-item self-report instrument, the Mental Health Peer Support Questionnaire (MHPSQ), to assess young adults’ perceived knowledge and skills in mental health peer support.

**Methods:**

We administered the questionnaire to 102 students who were trained as peer supporters, and 306 students who were not trained as peer supporters (denoted as *non-peer supporters*), in five college and vocational schools. Exploratory factor analysis and descriptive statistics were conducted. Cronbach’s α was used to assess reliability, and independent sample t-tests to assess criterion validity.

**Results:**

Exploratory factor analysis indicated a three-factor structure with adequate internal reliability (discerning stigma [α = .76], personal mastery [α = .77], skills in handling challenging interpersonal situations [α = .74]; overall scale [α = .74]). Consistent with establishing criterion validity, peer supporters rated themselves as significantly more knowledgeable and skilled than non-peer supporters on all items except two: (1) letting peer support recipients make their own mental health decisions, and (2) young adults’ self-awareness of feeling overwhelmed. Peer supporters who had served the role for a longer period of time had significantly higher perceived awareness of stigma affecting mental health help-seeking. Peer supporters who had reached out to more peer support recipients reported significantly higher perceived skills in handling challenging interpersonal situations, particularly in encouraging professional help-seeking and identifying warning signs of suicide.

**Conclusions:**

The MHPSQ may be a useful tool for obtaining a baseline assessment of young adults’ perceived knowledge and skills in mental health peer support, prior to them being trained as peer supporters. This could facilitate tailoring of training programs based on young adults’ initial understanding of mental health peer support. Subsequent to young adults’ training and application of skills, the MHPSQ could also be applied to evaluate the effectiveness of peer programs and mental health training.

**Supplementary Information:**

The online version contains supplementary material available at 10.1186/s13033-022-00555-6.

## Background

Mental illness is a growing public health concern, with roughly half of all lifetime mental disorders starting by the mid-teens and three-quarters by the mid-20s [24]. Young adults living with serious and distressing mental health problems may refuse to seek help or considerably delay seeking professional mental health services [7, 18], due to concerns such as stigma, embarrassment and confidentiality [19]. This may lead to adverse health outcomes, including substance abuse, risky sexual behavior, lower quality of adult life and premature death [3, 10, 11, 26]). More concerted efforts have to be conducted to improve the mental health well-being and help-seeking behaviors of young adults.

Peer support is an intervention that could benefit young adults facing emotional and mental health distress. Within the context of a school setting, peer support is defined as a process of assistance whereby trained, supervised students help other students with personal and school-related problems, offer supportive relationships, clarify peer support recipients’ (denoted as *recipients*) thoughts and feelings, explore options and help recipients in determining their own solutions [32]. In various countries, peer support programs have been extensively used to supplement traditional approaches to help college and vocational students, such as school counseling services [22]. Training and supervising young adults to reach their peers with health-related information have long been used to promote help-seeking behaviors [6].

In Singapore, young adults have the highest 12-month and lifetime prevalence of mental illness, as compared to older age groups [43]. A nation-wide survey found that only 6% of the population and 32% of those with depressive and anxiety disorders sought professional help for mental and emotional problems [33]. In 2016, an inter-governmental agency taskforce was formed in Singapore, charged to enhance young adults’ mental health. As part of this initiative, concerted efforts have been made to strengthen peer support networks for young adults in colleges and vocational schools throughout the country. This initiative requires all colleges and vocational schools in the country to set up a peer support program. Within each school, some of the students served as peer supporters and some were students who were not part of the peer support program (denoted as *non-peer supporters*). All schools provide training for peer supporters on topics such as mental health awareness, listening skills, and referring other students to professional resources, to help these students take on the role [29].

Despite the extensive literature on peer support programs for individuals with serious mental illness, few studies have investigated peer support programs for mental health promotion in colleges and vocational schools. Most studies on school-based mental health peer support were conducted in secondary or high schools [1, 13, 15, 20, 21, 38, 41] and one was conducted in a veterinary school [40]. Out of the four studies found that were conducted among college students, two had small sample sizes of 20 to 30 peer supporters [2, 4], one collected only qualitative data [4], and two did not collect any primary data [22, 42]. Only two studies on school-based programs were found to have collected data from recipients of these peer programs [1, 40]. One review was available regarding a peer program in the Asian context, but it did not involve any data collection [22]. No studies thus far have assessed the knowledge and skills of mental health peer support among young adults in Asia.

The purpose of this study was to develop and validate the Mental Health Peer Support Questionnaire (MHPSQ), an instrument intended to assess perceived mental health peer support knowledge and skills among young adults in college and vocational schools with peer support programs. We provide an exploratory factor analysis (EFA) on the underlying structure of the measured mental health peer support variables, and initial evidence of validity and reliability of the MHPSQ for potential use in other school-based peer support training programs.

As a check of criterion validity, we analyzed the responses from both peer supporters and non-peer supporters. We took into consideration whether participants had been trained to provide mental health peer support, and if they were, their self-reported peer support experiences. We hypothesized that participants who were trained to be peer supporters would have higher perceived knowledge of and skills in mental health peer support, relative to those who were not trained. A second hypothesis was that among peer supporters, having more experience in the role would be associated with greater perceived knowledge of and skills in mental health peer support.

The validated instrument could be used to obtain a baseline assessment of young adults’ understanding of mental health peer support prior to being trained, to facilitate the tailoring of peer support training programs. The instrument may also be used for the evaluation of peer support programs’ effectiveness subsequent to peer supporters’ training and practice.

## Methods

### Study sample

The collaborating institution partnering the schools to develop peer support programs, the Health Promotion Board Singapore, contacted eight colleges and vocational schools in Singapore that had an existing peer support program. Five of the eight schools agreed to participate in the study. To maintain confidentiality of the schools, the schools will be denoted in the order of how long their peer support program had been established, ranging from S1 (longest established program) to S5 (most recently established program).

Participating schools sent emails which contained a brief description of the study and links to the online surveys, to both peer supporters and non-peer supporters. Research team members also recruited additional non-peer supporters from public spaces (e.g., hallways, cafeterias, study rooms) of the five participating schools to invite them to participate in the online survey. Informed consent was obtained through the online survey platform, Qualtrics, before participants could proceed to complete the online surveys. Towels or stress balls were given as tokens of appreciation to participants who indicated interest in participating in the online survey. At the end of the study, each participating school received a report of their own schools’ data and students’ feedback on their program. The project was reviewed and approved by the Institutional Review Board of the Johns Hopkins University Bloomberg School of Public Health.

### Study procedures

The online survey for peer supporters, administered through Qualtrics, included questions about basic demographics, the participants’ level of experience in the peer support role and the MHPSQ. For non-peer supporters, the survey included questions about basic demographics and the MHPSQ.

### Measurement development

#### Mental Health Peer Support Questionnaire (MHPSQ)

As majority of the participating schools used the peer supporter training workshops provided by the Health Promotion Board, we referred to the training content of these peer supporter training workshops to identify major topics that peer supporter trainings had in common. Peer supporter training provided by all schools included content reflecting five common training topics: (1) fundamental skills of peer support, (2) self-care, (3) understanding mental health, (4) identifying distress in peers, and (5) promoting help-seeking behavior. A pool of items was generated that assessed perceived knowledge and skills under each of the common training topics.

We sought assistance from five consultants in the Health Promotion Board who were in charge of designing the program and collaborating with the schools. This was to ensure that the topics identified and the MHPSQ items generated were coordinated with what was taught to peer supporters in training programs throughout all the participating schools. We circulated drafts of the instrument to the consultants for comments in an iterative manner. Feedback was incorporated into final versions of the questionnaire, which enabled us to arrive at a smaller number of items. We also edited the item wording to better fit the students’ language and mental health literacy levels. The final version of the MHPSQ included 20 items. Responses to each item were evaluated on a six-point rating scale ranging from “Strongly disagree” (coded 1) to “Strongly agree” (coded 6). A six-point scale was used to avoid neutral responses while capturing more granular differences in rating.

#### Participant characteristics

The online surveys for both peer supporters and non-peer supporters gathered information on sociodemographic characteristics, such as age (in years), gender (male, female), self-identified race (Chinese, Malay, Indian, Caucasian, Other), school and graduation year (2019, 2020, 2021, 2022, Other). Age was recoded as a categorical variable due to small numbers in higher ages. Graduation year and school information were used to calculate the number of years students had spent in their school. Peer supporters were also asked questions to assess their level of experience as a peer supporter: (1) when they attended training workshop(s) to become a peer supporter (one-month intervals from < 1 month ago to > 12 months ago), (2) the number of recipients they had supported since their first training (None, 1 to 5, 6 to 10, 11 to 15, 16 to 20, > 20), (3) the number of recipients they recommended to seek professional help (None, 1 to 5, 6 to 10, 11 to 15, 16 to 20, > 20), and (4) the number of recipients who had sought professional help, among those whom the peer supporters had recommended to seek help (None, 1 to 5, 6 to 10, 11 to 15, 16 to 20, > 20, Don’t know).

### Analytic strategy

To assess validity and reliability of the MHPSQ, our data analysis proceeded in six phases. First, data were inspected for errors and outliers and cleaned as needed. Only participants who completed all 20 items of the instrument were retained in subsequent analyses. Second, exploratory factor analysis (EFA) for all items on the scale were carried out to identify underlying relations between measured mental health peer support variables. The EFA sample size meets the general guidelines of *N* = 200 and at least ten participants per indicator [31, 34]. Scree plots, eigenvalues and parallel analysis based on the 20 MHPSQ items were used to determine the number of factors to retain. Two eigenvalues were greater than 1, three eigenvalues were greater than 0.70. Scree plots suggested two factors while parallel analysis suggested three factors. We explored the possibility of two or three correlated factors accounting for the data. Maximum likelihood procedures were employed and all solutions were rotated obliquely using oblimin rotation [23]. Maximum likelihood allows for the computation of a wide range of goodness of fit indices, factor correlations and confidence interval computations [16]. Oblimin rotation is preferable when underlying factors could be correlated [36]. Pattern matrices were analyzed using 0.32 as the cutoff value for a salient factor loading, which corresponds to a minimum 10% overlap in variance with other items in the same factor [44]. Crossloading items that loaded at 0.32 or higher on more than one factor were dropped from the analysis. Third, in conducting descriptive data analyses, we investigated the distributional properties of each demographic (for both peer supporters and non-peer supporters) and experience (only for peer supporters) variable. Pearson’s chi-square [35] was used to identify any possible trend among responses that were missing or incomplete. Fourth, we computed Cronbach’s alpha coefficients [12] for the subscales. Fifth, we sorted the peer supporter sample into two reference groups based on when they had been trained, as a proxy for how long they had been active peer supporters in the program: above the median (more than eight months ago) and at or below the median (eight months or less). To assess criterion validity, we compared peer supporters above the median duration as active peer supporters to those below the median, based on individual items and total scores of the MHPSQ factors. We also sorted the peer supporter sample into two reference groups based on how many recipients they had supported since their first training: above the median (six or more recipients) and at or below the median (fewer than six recipients). We then conducted comparisons on individual items and total scores of the MHPSQ factors. We hypothesized that among peer supporters, having more experience in the role would be associated with higher perceived mental health knowledge and peer support skills. Sixth, to further assess criterion validity, we compared peer supporters with non-peer supporters, based on individual items and total scores of the MHPSQ factors. We also hypothesized that participants who were trained to be peer supporters would have greater perceived knowledge of and skills in mental health peer support, relative to those who were not trained. We used independent sample t-tests [25] to compare item and total factor mean scores between high and low experience groups and between peer supporters and non-peer supporters, with an α of 0.05 for statistical significance. All analyses were conducted with the R statistical program, version 3.5.1 [37].

## Results

### Sample characteristics

Of 148 peer supporters who started the peer supporter questionnaire, 102 completed all items on the MHPSQ (68.9% completion rate). There was no particular point in the questionnaire where attrition occurred. When demographic and experience covariates were examined, peer supporters who completed the MHPSQ (*n* = 101) and those who did not complete the MHPSQ (*n* = 14) were significantly different in terms of gender (χ^2^ (1, *n* = 115) = 4.57*, p* < 0.05) and race (χ^2^ (3, *n* = 115) = 9.42*, p* < 0.05). Fewer males and Indians completed the MHPSQ.

Of 536 non-peer supporters who started the non-peer supporter questionnaire, 306 completed all items of the MHPSQ (57.1% completion rate). There was also no particular point in the questionnaire where attrition occurred. No statistically significant demographic differences were found between non-peer supporters who completed the MHPSQ (*n* = 263) and those who did not complete the MHPSQ (*n* = 24), except for the school they were enrolled in (χ^2^ (4, *n* = 287) = 9.60*, p* < 0.05). S1 and S4 had fewer non-peer supporters completing the MHPSQ.

The peer supporter and non-peer supporter questionnaires took an average of 15.2 and 15.6 min to complete, respectively. This calculation excluded durations that exceeded 5.5 h, as those participants were likely to not have completed the survey at one sitting.

Table 1 shows the demographics of the analytic sample, the 102 peer supporters and 306 non-peer supporters who completed the MHPSQ. The mean ages of peer supporters and non-peer supporters were 20.6 years (*SD* = 2.61) and 20.7 years (*SD* = 2.55) respectively. The mean amounts of time peer supporters and non-peer supporters had been enrolled in their school were 1.34 years (*SD* = 1.26) and 1.13 years (*SD* = 1.30) respectively. Approximately 74.2% of Singapore’s population is ethnically Chinese [39], thus there was a slight overrepresentation of Chinese in our peer supporter sample (81.2%). More females participated in the peer supporter survey than males.

**Table 1:** Demographics of peer supporters and non-peer supporters, Singapore 2018

	Peer supporters	Non-peer supporters
	Total *n* (%)	Total *n* (%)
Demographic characteristics		
Age, in years		
18–20	53 (52.5)	138 (49.6)
21–24	39 (38.6)	120 (43.2)
25–30	9 (8.9)	20 (7.2)
Gender		
Male	35 (34.7)	143 (49.7)
Female	66 (65.3)	145 (50.3)
Race/Ethnicity		
Caucasian	0 (0)	3 (1.1)
Chinese	82 (81.2)	213 (74.7)
Indian	3 (3.0)	19 (6.7)
Malay	9 (8.9)	30 (10.5)
Other	7 (6.9)	20 (7.0)
School		
S1	35 (36.8)	63 (24.0)
S2	19 (20.0)	67 (25.5)
S3	19 (20.0)	40 (15.2)
S4	13 (13.7)	42 (16.0)
S5	9 (9.5)	51 (19.4)
Years in school		
< 1 year	35 (38.9)	124 (50.4)
1–2 years	37 (41.1)	82 (33.3)
3–4 years	18 (20.0)	40 (16.3)
Peer supporter experience		
Duration since attending training workshop		
≤ 4 months	33 (33.0)	–
5–8 months	18 (18.0)	–
≥ 9 months	49 (49.0)	–
Number of peers supported (since training)		
None	19 (18.6)	–
1 to 5	55 (53.9)	–
> 6	28 (27.5)	–
Number of peers recommended to seek professional help (since training)		
None	58 (56.9)	–
1 to 5	39 (38.2)	–
> 6	5 (4.9)	–
Number of peers, out of those recommended to seek professional help, actually sought help (since training)		
None	65 (71.4)	–
1 to 5	24 (26.4)	–
> 6	2 (2.2)	–

Number of students who submitted demographics data might not match the total number who completed the MHPSQ, due to incomplete or missing data

### Factor analysis

The three-factor solution (GFI = 0.92, AGFI = 0.87, RMSR = 0.03, RMSEA = 0.04, CFI = 0.97) had better model fit than the two-factor solution (GFI = 0.81, AGFI = 0.76, RMSR = 0.06, RMSEA = 0.07, CFI = 0.87). Further comparisons with alternative models are shown in the Additional file 1.

The three-factor solution with 16 items was used for subsequent analyses. The first factor represented items associated with discerning stereotypes and prejudices (denoted as *Discerning Stigma)* (1) “Young people with mental illness are dangerous;” (2) “People with mental illness can just get over their emotional problems if they try;” (3) “Serious mental health problems are obvious;” (4) “Stigma does not affect people’s willingness to seek help.” The second factor consisted of eight items related to personal mastery in one’s peer support abilities and judgment (*Personal Mastery*): (1) “Self-care is important;” (2) “Mental health stigma can be caused by media portrayals;” (3) “I am able to understand my peers with empathy;” (4) “I can talk about ways of coping with my peers;” (5) “I am confident of letting my peers make their own decisions about their mental health;” (6) “I am aware of when I am feeling overwhelmed;” (7) “I know the differences between self-harm and suicide;” (8) “I am able to encourage my peers to have positive mental health.” The third factor was associated with skills in handling challenging interpersonal situations (*Interpersonal Skills*): (1) “I find it difficult to encourage my peers to seek professional help when they need it;” (2) “I find it hard to tell my peers concerns in a non-judgmental way;” (3) “I tend to focus on myself in conversations with my peers, even when I don't mean to;” (4) “I find it difficult to identify warning signs of suicide.”

The three-factor model showed good fit (GFI = 0.92, AGFI = 0.87, RMSR = 0.03, RMSEA = 0.04, CFI = 0.97), acceptable internal reliability (α = 0.74) and cumulatively explained 36.9% of the variance. Factor loadings of the items, eigenvalues, percent of total variance explained and scale reliability values (Cronbach’s α coefficient) for each of the three factors are shown in Table 2. Factor correlations indicated divergent validity (correlation between Discerning Stigma and Personal Mastery = 0.06; correlation between Discerning Stigma and Interpersonal Skills = 0.50; correlation between Personal Mastery and Interpersonal Skills = 0.02).

**Table 2:** Summary of exploratory factor analysis results for the Mental Health Peer Support Questionnaire (MHPSQ), Singapore 2018

	Factor loadings		
	Discerning stigma (Factor 1)	Personal mastery (Factor 2)	Interpersonal skills (Factor 3)
Young people with mental illness are dangerous^a^	.**46**	.01	.17
People with mental illness can just get over their emotional problems if they try^a^	.**65**	.04	.13
Serious mental health problems are obvious^a^	.**69**	− .03	.00
Stigma does not affect people’s willingness to seek help^a^	.**68**	− .01	− .02
Self-care is important	.14	.**55**	.03
Mental health stigma can be caused by media portrayals	.23	.**41**	− .13
I am able to understand my peers with empathy	.12	.**67**	− .04
I can talk about ways of coping with my peers	-.17	.**63**	.06
I am confident of letting my peers make their own decisions about their mental health	-.15	.**37**	− .11
I am aware of when I am feeling overwhelmed	.06	.**60**	− .05
I know the differences between self-harm and suicide	− .04	.**53**	.05
I am able to encourage my peers to have positive mental health	− .12	.**63**	.04
I find it difficult to encourage my peers to seek professional help when they need it^a^	− .03	− .01	.**65**
I find it hard to tell my peers concerns in a non-judgmental way^a^	.04	.04	.**64**
I tend to focus on myself in conversations with my peers, even when I don't mean to^a^	.11	− .02	.**60**
I find it difficult to identify warning signs of suicide^a^	− .01	.00	.**62**
Eigenvalues	1.84	2.49	1.75
Percent of variance	11.0	15.5	10.4
Cronbach’s α (95% CI)	.76 (.72, .79)	.77 (.73, .80)	.74 (.70, .78)

Only factor loadings greater than .32 are presented

^a^Items were reverse scored in the questionnaire and for the factor analysis

Bold values indicate salient factor loading for each item

### Criterion validity

#### Comparison among peer supporters, by experience with peer support

Mean ratings for each item and total factor scores were calculated and compared between peer supporters that reported having been active peer supporters for a period above the sample median (calculated based on when they were trained; *median* = 8 months), and those that were at or below the median. For the Discerning Stigma factor, the reverse-coded item “Stigma does not affect people’s willingness to seek help” (*M* = 0.58, 95% CI = [0.04, 1.12], *p* < 0.05) was significantly higher for peer supporters who reported having been active for a longer period of time. There were no statistically significant differences in total factor and item scores for the Personal Mastery and Interpersonal Skills factors.

The same comparison was conducted between peer supporters that reported having supported a number of recipients above the sample median (one to five recipients), and those who supported a number of recipients at or below the median. There were no statistically significant differences in total factor and item scores for Discerning Stigma and Personal Mastery factors. For the Interpersonal Skills factor, the reverse-coded items “I find it difficult to encourage my peers to seek professional help when they need it” (*M* = 0.70, 95% CI = [0.18, 1.22], *p* < 0.01) and “I find it difficult to identify warning signs of suicide” (*M* = 0.56, 95% CI = [0.06, 1.03], *p* < 0.05) were significantly higher for peer supporters who reported having supported more recipients. The total score for Interpersonal Skills was also significantly higher (*M* = 1.67, 95% CI = [0.09, 3.25], *p* < 0.05) for peer supporters who reported having supported more recipients.

#### Comparison between peer supporters and non-peer supporters

We calculated and compared each item and total factor scores between peer supporters and non-peer supporters (Table 3). Peer supporters had significantly higher mean ratings of total factor and all item scores for all three factors, except for the items “I am confident of letting my peers make their own decisions about their mental health” and “I am aware of when I am feeling overwhelmed” in the Personal Mastery factor.

**Table 3:** Results of T-Test and descriptive statistics comparing peer supporters and non-peer supporters for the Mental Health Peer Support Questionnaire (MHPSQ), Singapore 2018

	Peer supporters			Non-peer supporters			95% CI for Mean Difference		
	M	SD	n	M	SD	n		t	df
**Discerning** **stigma**									
Young people with mental illness are dangerous^a^	4.15	1.42	102	3.14	1.44	306	(.68, 1.33)***	6.12	406
People with mental illness can just get over their emotional problems if they try^a^	4.34	1.38	102	3.75	1.46	306	(.27, 0.92)***	3.62	406
Serious mental health problems are obvious^a^	3.90	1.31	102	3.32	1.47	306	(.26, 0.90)***	3.56	406
Stigma does not affect people’s willingness to seek help^a^	4.51	1.40	102	3.67	1.49	306	(.51, 1.17)***	5.00	406
*Total score*	16.90	3.97	102	13.88	4.41	306	(2.05, 3.99)***	6.13	406
**Personal mastery**									
Self-care is important	5.61	.60	102	5.32	.86	306	(.14, .44)***	3.73	247.75
Mental health stigma can be caused by media portrayals	5.00	.89	102	4.68	1.02	306	(.09, .54)**	2.79	406
I am able to understand my peers with empathy	4.92	.78	102	4.67	.92	306	(.07, .44)**	2.69	202.73
I can talk about ways of coping with my peers	4.87	.71	102	4.43	.99	306	(.26, .62)***	4.87	240.67
I am confident of letting my peers make their own decisions about their mental health	4.36	.93	102	4.29	.93	306	(-.14, .28)	.68	406
I am aware of when I am feeling overwhelmed	4.95	.92	102	4.77	.93	306	(-.03, .39)	1.66	406
I know the differences between self-harm and suicide	5.10	.91	102	4.72	1.05	306	(.15, .61)**	3.26	406
I am able to encourage my peers to have positive mental health	4.84	.74	102	4.47	.91	306	(.20, .55)***	4.18	209.37
*Total score*	39.66	3.56	102	37.36	4.76	306	(1.42, 3.18)***	5.17	230.43
**Interpersonal skills**									
I find it difficult to encourage my peers to seek professional help when they need it ^a^	3.49	1.22	102	3.08	1.16	306	(.14, .67)**	3.02	406
I find it hard to tell my peers concerns in a non-judgmental way ^a^	3.83	1.15	102	3.12	1.16	306	(.45, .97)***	5.39	406
I tend to focus on myself in conversations with my peers, even when I don't mean to ^a^	4.04	1.36	102	3.22	1.12	306	(.52, 1.11)***	5.49	149.60
I find it difficult to identify warning signs of suicide ^a^	3.57	1.12	102	3.00	1.15	306	(.31, .83)***	4.37	406
*Total score*	14.93	3.64	102	12.42	3.33	306	(1.74, 3.27)***	6.42	406

*p < .05. **p < .01. ***p < .001

^a^Items were reverse scored in the questionnaire and for the analysis. Larger values indicate higher perceived knowledge/skills

## Discussion

### Overview

The purpose of this study was to develop and validate an instrument for measuring the perceived knowledge of and skills in mental health peer support among young adults in colleges and vocational schools in Singapore. In the current study, EFA was used to identify underlying relations among measured variables on mental health peer support. EFA uncovered three domains: discerning stereotypes and prejudices (*Discerning Stigma*), personal mastery in one’s abilities and judgment (*Personal Mastery*), and skills in handling challenging interpersonal situations (*Interpersonal Skills*). These three domains differed from the original five training topics that were used to guide the instrument design, suggesting that the latent constructs for mental health peer support centered around young adults’ perception of relative ease and challenges in applying mental health support to self and others in practice, and not how the training content was organized by consultants. Each of three identified factors had high internal reliability and criterion validity. As part of establishing criterion validity, our study also compared peer supporters across experience levels, and peer supporters to non-peer supporters. We hypothesized that peer supporters with more experience would have more perceived mental health knowledge and peer support skills than peer supporters with less experience, and that peer supporters would have more perceived knowledge and skills than non-peer supporters.

### Criterion validity

#### Relation between being a peer supporter and perceived mental health peer support knowledge and skills

Our study found that peer supporters, as compared to non-peer supporters, were significantly more likely to have higher perceived ability to discern stigma, higher perceived mastery of peer support abilities and judgment, and higher perceived skills in handling challenging interpersonal situations. These findings reveal robust criterion validity between being a peer supporter and almost all aspects of perceived peer support knowledge and skills, as all items were significantly different between peer supporters and non-peer supporters, except perceived confidence in letting peers make their own mental health decisions and perceived awareness of feeling overwhelmed. This could possibly be due to deficits in those specific areas of training or those concepts being more resistant to change through training. In other words, despite having received training on respecting their recipients’ mental health decisions, peer supporters could still believe that it would be better for their recipients’ long-term wellbeing if their recipients were more strongly encouraged to seek professional mental health help. Young adults may also either not require training to know when they are feeling burnt out, or the training was not effective in improving this skill. Those who were selected or volunteered to be peer supporters could also be better at self-care, thus less likely to feel overwhelmed regardless of training.

These results suggest that young adults’ perceived mental health knowledge and peer support skills were positively associated with experience as a peer supporter. Strong self-efficacy has been demonstrated to motivate and sustain one’s endeavors for optimal performance, increasing one’s attention and efforts to situational demands and resilience in the face of obstacles [5]. Our results suggest that peer supporter trainings could build young adults’ self-efficacy through increasing perceived knowledge and skills on specific domains covered in the MHPSQ, thus possibly increasing their motivations to be a positive pillar of support for their peers. In the peer support literature, studies found that training programs increased peer supporters’ recognition of suicide symptoms [21] and decreased their stigmatizing beliefs [20]. Peer support training also had positive impacts on general communication skills [40, 41] and specific empathetic and reflection skills [2, 9, 28]. Peer supporters had also reported self-development [40], increased confidence in supporting peers [15, 20], decreased nervousness and increased self-awareness [1]. A longitudinal study on secondary school peer supporters’ pre- and post-peer program participation found significant improvement in peer support skills and understanding [15], which corroborates the results from our study. These findings suggest that school-based programs that involve young adults in mental health training and supporting their peers could be an effective way to increase young adults’ perceived mental health literacy and skills to cope with their own and their peers’ mental health issues.

#### Relation between peer support experience and perceived mental health peer support knowledge and skills

We also found that young adults who had been peer supporters for a longer period of time were significantly more likely to recognize that stigma could affect one’s willingness to seek help, than those who had been peer supporters for a shorter duration. Peer supporters who had supported more peers reported significantly higher perceived confidence in encouraging their peers to seek professional help when necessary, in identifying warning signs of suicide, and in handling challenging interpersonal situations as a whole. These self-rated results indicate criterion validity between peer supporter experience and several components of perceived peer support knowledge and skills.

Supporting more recipients was associated with significantly increased perceived skills in handling a variety of challenging interpersonal situations, while being peer supporters for a longer period of time was not significantly associated with increased perceived skills. Therefore, these results suggest that the practical application of peer support skills through interaction with recipients is more strongly associated with increased perceived ability in handling challenges, relative to merely having been in the peer support program for a longer period of time. The findings also suggest that years of experience and real-life application of peer support skills might be more salient than the initial training in improving perceived knowledge and skills. This suggests that conducting ongoing trainings and sharing expertise among peer supporters could be beneficial program components that would facilitate knowledge and skill transfers.

### Study limitations

Before discussing the implications of our results, the limitations of our study should be considered. First, we were not able to collect longitudinal data comparing peer supporters pre- and post-training and after a follow-up period. Therefore, we were unable to determine that the peer support training and/or program had a causal effect on improving perceived mental health knowledge and skills. Nevertheless, we had a comparison group of students who did not participate in the peer support program, which allowed us to examine the validity of our instrument and the effectiveness of these programs. We also made an initial assessment of criterion validity by comparing self-ratings among peer supporters based on their experience level, and we found that peer supporters with more experience reported higher self-ratings in perceived knowledge and skills. Further studies should be conducted to determine whether the peer support training and/or experience improved students’ mental health knowledge and skills, or students with higher perceived knowledge and skills were more likely to volunteer or be nominated to join peer support programs. Future studies can administer our instrument to peer supporters pre- and post-training and at follow-up, to investigate whether peer support training or experience plays a larger role in improving perceived mental health knowledge and skills. Second, sample size limitations precluded our ability to conduct confirmatory factor analysis (CFA) to further verify the factor structure uncovered by EFA. A goal of future research would be to apply the instrument to a separate sample to test the extent the proposed factor structure could be replicated. Other knowledge and skill measures can also be applied to examine convergent validity of the instrument. Third, the assessment of perceived peer support knowledge and skills was based on self-reports, and young adults’ actual peer support practices could not be objectively verified. While the present measures are able to assess the self-efficacy young adults bring to the peer support role, future research can supplement self-ratings with observational studies or school counsellors’ assessment of young adults’ mental health knowledge and peer support skills. Third, despite our sample size being larger than most existing studies on peer support, the current study only involved colleges and vocational schools in Singapore. Thus, our results might not be generalizable to different cultures and education systems, and the assessment instrument should be tested with other samples. School-based peer support programs for young adults deserve attention because of the limited literature available on this topic, especially among countries in Asia. Our results from the Singapore context offer a unique perspective bridging Western and Eastern cultures, as Singapore’s culture and education system have been largely influenced by both the East and the West. This could facilitate the adaptability of this questionnaire to schools in other countries.

### Implications

The implications of school-based peer support research for public mental health are timely. Critiques of peer support programs have been centered on the program development and implementation procedures, and the program evaluation research methods. Major critiques of these programs highlighted problems in providing adequate peer supporter training and supervision, and underlined challenges in defining and limiting peer supporter roles [8, 14, 17, 27, 30, 32]. Main criticisms of research evaluating these programs had centered around the predominant use of qualitative methods, where conclusions were mainly based on subjective comments and difficult to replicate [14, 30, 41]. The success of peer support programs and peer supporters is largely contingent on peer supporters’ skill development through training and experience in the programs. However, very little in the way of quantitatively assessing peer support programs has been reported, and there is a need for an instrument can serve as a guide for peer supporter training and program evaluation. Our study offers a validated self-assessment instrument, the MHPSQ, that can be administered alongside peer support training to measure young adults’ mental health understanding and peer support skills. The MHPSQ may be used to quantitatively assess peer supporter training and young adults’ skill development with increasing peer support experience. In the case of program development, the instrument could be used to assess peer supporters’ baseline perceived mental health knowledge and skills, and to guide and tailor training programs based on peer supporters’ self-identified needs.

## Conclusions

Based on our sample, we have developed an instrument measuring core components of peer support programs, with demonstrated reliability and validity in a nation-wide peer support initiative. The availability of the MHPSQ as a brief assessment tool can facilitate the design and evaluation of school-based mental health peer support programs. A goal of future research would be to use CFA and qualitative methods to refine the instrument items, to further validate and improve the tool across various implementation contexts.

## Supplementary Information

**Additional file 1.** EFA model comparison statistics.

## Data Availability

The datasets analysed during this study are available from the corresponding author on reasonable request.

## Abbreviations

MHPSQ: Mental Health Peer Support Questionnaire
EFA: Exploratory factor analysis
CFA: Confirmatory factor analysis

## References

[1] Abu-Rasain MHM, Williams DI (1999). Peer counselling in Saudi Arabia. J Adolesc.

[2] Aladag M, Tezer E (2009). Effects of a peer helping training program on helping skills and self-growth of peer helpers. Hague.

[3] Anderson JE, Lowen CA (2010). Connecting youth with health services: Systematic review. Can Fam Physician.

[4] Badura AS, Millard M, Johnson C, Stewart A, Bartolomei S. Positive outcomes of volunteering as a peer educator: A qualitative study (Report No. CG 032-187). Creighton University. (ERIC Document Reproduction Service No. ED 473426). Retrieved from EBSCOHost ERIC database. 2003

[5] Bandura A (1982). Self-efficacy mechanism in human agency. Am Psychol.

[6] Barker G (2007). Adolescents, social support and help-seeking behaviour: an international literature review and programme consultation with recommendations for action.

[7] Biddle L, Donovan JL, Gunnell D, Sharp D (2006). Young adults' perceptions of GPs as a help source for mental distress: a qualitative study. Br J Gen Pract.

[8] Black DR, Tobler NS, Sciacca JP (1998). Peer helping/involvement: an efficacious way to meet the challenge of reducing alcohol, tobacco, and other drug use among youth?. J Sch Health.

[9] Brenton, Kelly Lynn . Report of a counselling internship at Eugene Vaters Academy and Junior High, St. John's, Newfoundland, including a research project: implementation and evaluation of the effectiveness of a peer helping training program at the Junior High. Masters thesis, Memorial University of Newfoundland, 1999

[10] Brindis C, Hair E, Cochran S, Cleveland K, Valderrama L, Park M (2007). Increasing access to program information: A strategy for improving adolescent health. Matern Child Health J.

[11] Brindis C, Park MJ, Ozer EM, Irwin J, Charles E (2002). Adolescents' access to health services and clinical preventive health care: Crossing the great divide. Pediatr Ann.

[12] Cronbach LJ (1951). Coefficient alpha and the internal structure of tests. Psychometrika.

[13] Diver-Stamnes AC (1991). Assessing the effectiveness of an inner-city high school peer counseling program. Urban Educ.

[14] Downe AG, Altmann HA, Nysetvold I (1986). Peer counseling: More on an emerging strategy. Sch Couns.

[15] Eisenstein C, Zamperoni V, Humphrey N, Deighton J, Wolpert M, Rosan C (2019). Evaluating the peer education project in secondary schools. J Public Ment Health.

[16] Fabrigar LR, Wegener DT, MacCallum RC, Strahan EJ (1999). Evaluating the use of exploratory factor analysis in psychological research. Psychol Methods.

[17] Fennell R (1993). A review of evaluations of peer education programs. J Am Coll Health.

[18] Goodman R, Ford T, Meltzer H (2002). Mental health problems of children in the community: 18 month follow up. BMJ.

[19] Gulliver A, Griffiths KM, Christensen H (2010). Perceived barriers and facilitators to mental health help-seeking in young people: a systematic review. BMC Psychiatry.

[20] Hart LM, Morgan AJ, Rossetto A, Kelly CM, Mackinnon A, Jorm AF (2018). Helping adolescents to better support their peers with a mental health problem: a cluster-randomised crossover trial of teen mental health first aid. Aust N Z J Psychiatry.

[21] Hennig CW, Crabtree CR, Baum D (1998). Mental health CPR: Peer contracting as a response to potential suicide in adolescents. Arch Suicide Res.

[22] Hsi T, Chung YLA. Peer helpers: Bridging the gap between the student community and the university’s counseling service. Paper presented at the 12th Asia--Pacific Student Services Association Conference 2010. 2010.

[23] Jackson JE, Armitage P, Colton T (2005). Oblimin rotation. Encyclopedia of biostatistics.

[24] Kessler R, Amminger G, Aguilar-Gaxiola S, Alonso J, Lee S, Üstün T (2007). Age of onset of mental disorders: a review of recent literature. Curr Opin Psychiatry.

[25] Kim TK (2015). T test as a parametric statistic. Korean J Anesthesiol.

[26] Laski L (2015). Realising the health and wellbeing of adolescents. BMJ.

[27] Lewis MW, Lewis AC (1996). Peer helping programs: helper role, supervisor training, and suicidal behavior. J Couns Dev.

[28] Martin AA, II. The effectiveness of Christian adolescent peer counselor training: a controlled study. Available from ProQuest Dissertations & Theses Full text: the humanities and social sciences collection. 1998.

[29] Ministry of Health Singapore, Ministry of Education Singapore (2016). NurtureSG action plan report.

[30] Morey RE, Miller CD, Rosén LA, Fulton R (1993). High school peer counseling: the relationship between student satisfaction and peer counselors' style of helping. Sch Couns.

[31] Myers ND, Ahn S, Jin Y (2011). Sample size and power estimates for a confirmatory factor analytic model in exercise and sport: a Monte Carlo approach. Res Q Exerc Sport.

[32] Myrick RD, Folk BE (1999). The power of peervention: a manual for the trainers of peer facilitators.

[33] Ng TP, Jin AZ, Ho R, Chua HC, Fones CSL, Lim L (2008). Health beliefs and help seeking for depressive and anxiety disorders among urban Singaporean adults. Psychiatr Serv.

[34] Norman GR, Streiner DL (2014). Biostatistics: the bare essentials.

[35] Pearson K (1990). On the criterion that a given system of deviations from the probable in the case of a correlated system of variables is such that it can be reasonably supposed to have arisen from random sampling. Philos Mag Series.

[36] Pett MA, Lackey NR, Sullivan JJ (2003). Making sense of factor analysis: the use of factor analysis for instrument development in health care research.

[37] R CoreTeam (2018). R: A language and environment for statistical computing.

[38] Robinson SE, Morrow S, Kigin T, Lindeman M (1991). Peer counselors in a high school setting: Evaluation of training and impact on students. Sch Couns.

[39] Singapore population. 2018. http://worldpopulationreview.com/countries/singapore-population/. Accessed 13 Dec 2018.

[40] Spielman S, Hughes K, Rhind S (2015). Development, evaluation, and evolution of a peer support program in veterinary medical education. J Vet Med Educ.

[41] Steinbauer L. The effects of a high school peer counselor training program on the self-appraisal skills and the interpersonal skills of the trained peer leader. ProQuest Dissertations & Theses Full text: the humanities and social sciences collection. 1998.

[42] Stokes DR, Gonzales M, Rowe D, Romero D, Adams M, Lyons S, Rayfield GE (1988). Multicultural peer counseling: a developmental perspective and rationale. J Couns Dev.

[43] Subramaniam M, Abdin E, Vaingankar JA, Shafie S, Chua BY, Sambasivam R (2019). Tracking the mental health of a nation: prevalence and correlates of mental disorders in the second Singapore Mental Health Study. Epidemiol Psychiatr Sci..

[44] Tabachnick BG, Fidell LS (2001). Using multivariate statistics.

