# Supplementary material for: Development of the Mental Health Peer Support Questionnaire in colleges and vocational schools in Singapore
Source: Int J Ment Health Syst. 2022 Sep 2;16:45. doi: 10.1186/s13033-022-00555-6 (PMC9438123; doi:10.1186/s13033-022-00555-6)
Supplement: Supplementary file 1 — Additional file 1. EFA model comparison statistics. [file 13033_2022_555_MOESM1_ESM.docx]

**Additional file 1: Author’s response to reviewer’s comment**

**Please consider performing EFA for peer supporters and non-peer supporters separately, because your sample was heterogenous when taking into account these two categories of participants. You can conduct EFA for a sample of 102 participants as some authors allow samples five times greater than the number of items and/or at least 100 participants as a prerequisite for doing EFA.**

We acknowledge the merits of the point raised by the reviewer.

As the purpose of the study was to develop and validate the Mental Health Peer Support Questionnaire (MHPSQ) for young adults, regardless of their level of peer support experience, we intended for the scale to be developed from and applicable to both peer supporters and non-peer supporters. This will allow the instrument to be used to assess young adults’ mental health understanding and peer support skills at different stages of the peer support program process, such as pre-peer support training/baseline prior to peer supporter selection (where we could expect the young adult to be similar to the non-peer supporter cohort), and post-peer support training (where the young adult could be more similar to the peer supporter cohort). With this intention in mind, performing the EFA separately for peer supporters and non-peer supporters could introduce biases in the psychometric development.

Nonetheless, we explored performing EFA for peer supporters and non-peer supporters separately. For both subsamples, two eigenvalues were greater than 1; three eigenvalues were greater than .70; scree plots suggested two factors while parallel analysis suggested three factors. Thus for both subsamples, we explored the possibility of two or three correlated factors accounting for the data. The EFA models and their statistics are summarized in Table A below.

Model A, the model currently used for subsequent analyses within our manuscript, remains as the best fitting model with the highest internal reliability. We would like to highlight that regardless of the sample selected, the three-factor solution performed better than the two-factor solution. Inspection of the factor loadings for all three-factor solutions (Models A, C, E) showed that throughout all three models, each item loaded consistently on the same factors. Thus, our conclusion of a three-factor model (Discerning Stigma, Personal Mastery, and Interpersonal Skills) would not be altered regardless of the sample chosen.

Given lesser relevance to our study’s objectives and little impact on the study’s overall conclusions, we do not think it would be necessary to report further on Models C to F in our manuscript.

Table A: EFA model comparison statistics

| **EFA models** | **# items** | **GFI** | **AGFI** | **RMSR** | **RMSEA** | **CFI** | **Cronbach’s α** |
| --- | --- | --- | --- | --- | --- | --- | --- |
| **Model A.** Three-factor solution, combined peer supporters and non-peer supporters sample (included in main text) | 16 | .92 | .87 | .03 | .04 | .97 | .74 |
| **Model B.** Two-factor solution, combined peer supporters and non-peer supporters sample (included in main text) | 18 | .81 | .76 | .06 | .07 | .87 | .75 |
| **Model C.** Three-factor solution, peer supporters only | 15 | .82 | .70 | .05 | .03 | .98 | .72 |
| **Model D.** Two-factor solution, peer supporters only | 17 | .63 | .52 | .08 | .08 | .79 | .74 |
| **Model E.** Three-factor solution, non-peer supporters only | 16 | .91 | .86 | .03 | .04 | .97 | .60 |
| **Model F.** Two-factor solution, non-peer supporters only | 19 | .80 | .74 | .06 | .07 | .87 | .64 |
